# Environmental justice burden and Black-White disparities in spontaneous preterm birth in Harris County, Texas

**DOI:** 10.3389/frph.2023.1296590

**Published:** 2023-12-19

**Authors:** K. W. Whitworth, I. Moussa, H. M. Salihu, A. Chardon Fabien, M. Suter, K. M. Aagaard, E. Symanski

**Affiliations:** ^1^ Section of Epidemiology and Population Sciences, Department of Medicine, Baylor College of Medicine, Houston, TX, United States; ^2^ Center for Precision Environmental Health, Baylor College of Medicine, Houston, TX, United States; ^3^ Department of Family & Community Medicine, Baylor College of Medicine, Houston, TX, United States; ^4^ Department of Obstetrics & Gynecology, Division of Maternal-Fetal Medicine, Baylor College of Medicine & Texas Children’s Hospital, Houston, TX, United States

**Keywords:** environmental justice, neighborhood, socioeconomic deprivation, preterm birth, health disparities, racial disparities

## Abstract

**Introduction:**

Given limited evidence of previous studies, we evaluated the role of environmental justice (EJ) burden (i.e., a neighborhood characterized by both increased environmental burden and socioeconomic deprivation) in Black-White disparities in spontaneous preterm birth (sPTB) in Harris County, Texas and compared results that evaluated neighborhood-level socioeconomic deprivation alone.

**Methods:**

We conducted a retrospective analysis using PeriBank, a database and biospecimen repository of gravidae giving birth at two hospitals in the Texas Medical Center. We included 3,703 non-Hispanic Black and 5,475 non-Hispanic white gravidae who were U.S.-born, delivered from August 2011-December 2020, and resided in Harris County, TX. We used data from the U.S. EPA EJScreen to characterize the EJ burden of participant's zip code of residence from fine particulate matter (PM_2.5_), ozone, and proximity to National Priorities List (NPL) sites and calculated zip-code level Area Deprivation Index (ADI). We assessed the contribution of neighborhood-level variables to the Black-White disparity in sPTB by evaluating attenuation of the odds ratio (OR) representing the effect of race in multivariable logistic regression models, controlling for individual-level characteristics. We also conducted race-stratified analyses between each neighborhood variable and sPTB. Exposure indices were treated as continuous variables; in stratified models, ORs and 95% Confidence Intervals (CIs) are presented per 10-unit increase in the neighborhood variable.

**Results:**

Accounting for individual-level variables, Black gravidae had 79% higher odds of sPTB than white gravidae (OR = 1.79, 95%CI = 1.32, 2.44); the disparity was moderately attenuated when accounting for EJ burden or ADI (ORs ranged from 1.58 to 1.69). Though we observed no association between any of the EJ burden indices and sPTB among white gravidae, we found increased risks among Black gravidae, with ORs of similar magnitude for each EJ variable. For example, Black gravidae experienced 17% increased odds of sPTB associated with a 10-unit increase in the EJ burden index for PM_2.5_ (OR = 1.17, 95%CI = 0.97, 1.40). No racial differences were observed in the association of ADI with sPTB.

**Discussion:**

Though we observed limited evidence of the contribution of living in EJ neighborhoods to the Black-White disparity in sPTB, our study suggests living in an EJ neighborhood may differentially impact Black and white gravidae.

## 1. Introduction

Preterm birth (i.e., the delivery of a neonate prior to 37 weeks gestation) has broad economic and social implications, for both maternal and newborn health. The economic burden of preterm birth in the United States (U.S.) was estimated to be more than $25 billion in 2016; while the majority of these costs were associated with newborn and early childhood medical care, more than $5 billion was attributed to lost productivity in adulthood (1). Pregnant gravidae who deliver a preterm neonate are more likely than their counterparts to develop significant medical co-morbidities later in life, particularly cardiac complications, and preterm neonates are at increased risk of complications ranging from neurologic deficits to pulmonary, cardiac, or metabolic disorders (2–5). Moreover, preterm birth is not borne equally among racial and ethnic groups in the U.S. In 2018, the prevalence of preterm birth among non-Hispanic Black gravidae in this country was 14.1% compared with 9.1% among non-Hispanic white gravidae; further, the prevalence of preterm birth among Black individuals is increasing at a steeper rate than their white counterparts (6) and the burden of the Black-White preterm birth disparity is largely limited to gravidae born in the U.S (7).

Though there are a number of individual-level risk factors associated with the occurrence of preterm birth including maternal age, marital status, parity, maternal smoking, and access to healthcare, these factors alone do not explain the majority of the observed Black-White disparity in prevalence of preterm birth in the U.S (8–13). Thus, there is a need to consider the broader social context within which women live. Following the Ecosocial Theory as outlined by Krieger (14), social inequalities in health and wellbeing are embodied through simultaneous and diverse routes involving, for example, exposure to social inequality and economic deprivation, exogenous hazards (e.g., environmental chemical exposures), and historic trauma. Stressors in the neighborhood environment activate the hypothalamic pituitary adrenal (HPA) axis (15), resulting in release of cortisol that can cross the placenta and adversely impact pregnancy (16, 17). This stress response provides a potential route through which neighborhood features may impact preterm birth risk and potentially mediate racial disparities in preterm birth.

It is also possible that, due to systemic and structural racism, Black and white women “embody” the neighborhood context differently, providing a pathway for race to modify associations between neighborhood contextual factors. Previous U.S.-based studies have attempted to quantify maternal risks associated with living in a socioeconomically deprived neighborhood though results have been mixed (18–21). Further, previous reviews of the U.S.-based literature provide evidence that this association may vary by maternal race (18, 19, 21). In the most recent meta-analysis of the topic published in 2016, Ncube et al. (19) reported a 27% increased risk of preterm birth associated with living in the most socioeconomically deprived neighborhoods compared with the least deprived neighborhoods [odds ratio (OR) = 1.27, 95% confidence interval (CI) = 1.16, 1.39]. However, there was no association between neighborhood socioeconomic deprivation and preterm birth for the sub-set of studies that adjusted for race (OR = 1.01, 95% CI = 0.94, 1.09). Further, this meta-analysis found that the magnitude of ORs representing associations between living in neighborhoods with higher vs. lower levels of socioeconomic deprivation was greater among white (OR = 1.61, 95% CI = 1.30, 2.00) than Black gravidae (OR = 1.15, 95% CI = 1.09, 1.21).

Beyond socioeconomic deprivation, other features of the neighborhood, such as environmental toxicant exposures like air pollution, may adversely impact perinatal health (22, 23). Unfortunately, some communities are doubly burdened by both socioeconomic disadvantage and environmental exposures and are deemed environmental justice (EJ) communities. Hence, measures of neighborhood-level environmental exposure alone do not fully capture the dual dimensions of EJ. In an analysis applying formal decomposition methods, zip-code level air pollution exposure provided only a modest contribution to observed racial disparities in preterm birth in California, pointing to the need to evaluate the impact of the neighborhood context beyond environmental exposure burden to further our understanding of key drivers of racial differences in perinatal health outcomes (10). Yet, few studies, to our knowledge, have explored the impact of living in an EJ communty (i.e., a neighborhood characterized not only by socioeconomic deprivation but also by increased environmental burden) on racial disparities in preterm birth and those investigations have reported equivocal findings. In one study using data for the period 2000–2005, inverse associations were reported between county-level prevalence of preterm birth and county-level environmental quality (considering factors related to both environmental contamination and socioeconomic deprivation) [Rappazzo et al. (24)]. In contrast, a more recent investigation using data from the U.S-based ECHO Cohort found moderately increased odds of preterm birth associated with living in a census tract with higher combined burden of environmental and social stressors (25). Moreover, in a stratified analysis, the association persisted only for Black women, suggesting that living in an EJ neighborhood may differentially impact risk of preterm birth among Black and white women.

Given equivocal and limited evidence of previous studies, we aimed to evaluate the role of EJ burden in Black-White disparities in preterm birth in Harris County, Texas, home to the fourth largest and most diverse city in the U.S. (Houston, TX). A secondary objective was to compare results to the impact of neighborhood-level socioeconomic deprivation alone.

## 2. Methods

### 2.1. Study population

We conducted a retrospective data analysis of deidentified data from gravidae enrolled in PeriBank, an IRB-approved perinatal database and biospecimen repository maintained by trained full-time research coordinators at Baylor College of Medicine in Houston, Texas. All gravid patients who are at least 18 years of age (or at least 16 years of age if emancipated) who deliver at our two institutional hospitals (Ben Taub Hospital and Texas Children's Pavilion for Women) are approached and offered participation in PeriBank, which began recruitment on August 1, 2011 (26). The rate of enrollment into PeriBank among qualified patients has not changed significantly with consent rates ranging from 86% to 90% over the study interval. Regular quarterly audits are done to ensure data accuracy. Maternal sociodemographic characteristics, zip codes in which gravidae lived and worked during the preconception period and in the 1st and 2nd/3rd trimesters during pregnancy, comorbidities, previous pregnancy history, and delivery data are collected and stored in PeriBank via abstraction of electronic medical records and participant interviews. PeriBank and the current study were approved by the Baylor College of Medicine Institutional Review Board.

**Figure 1:**
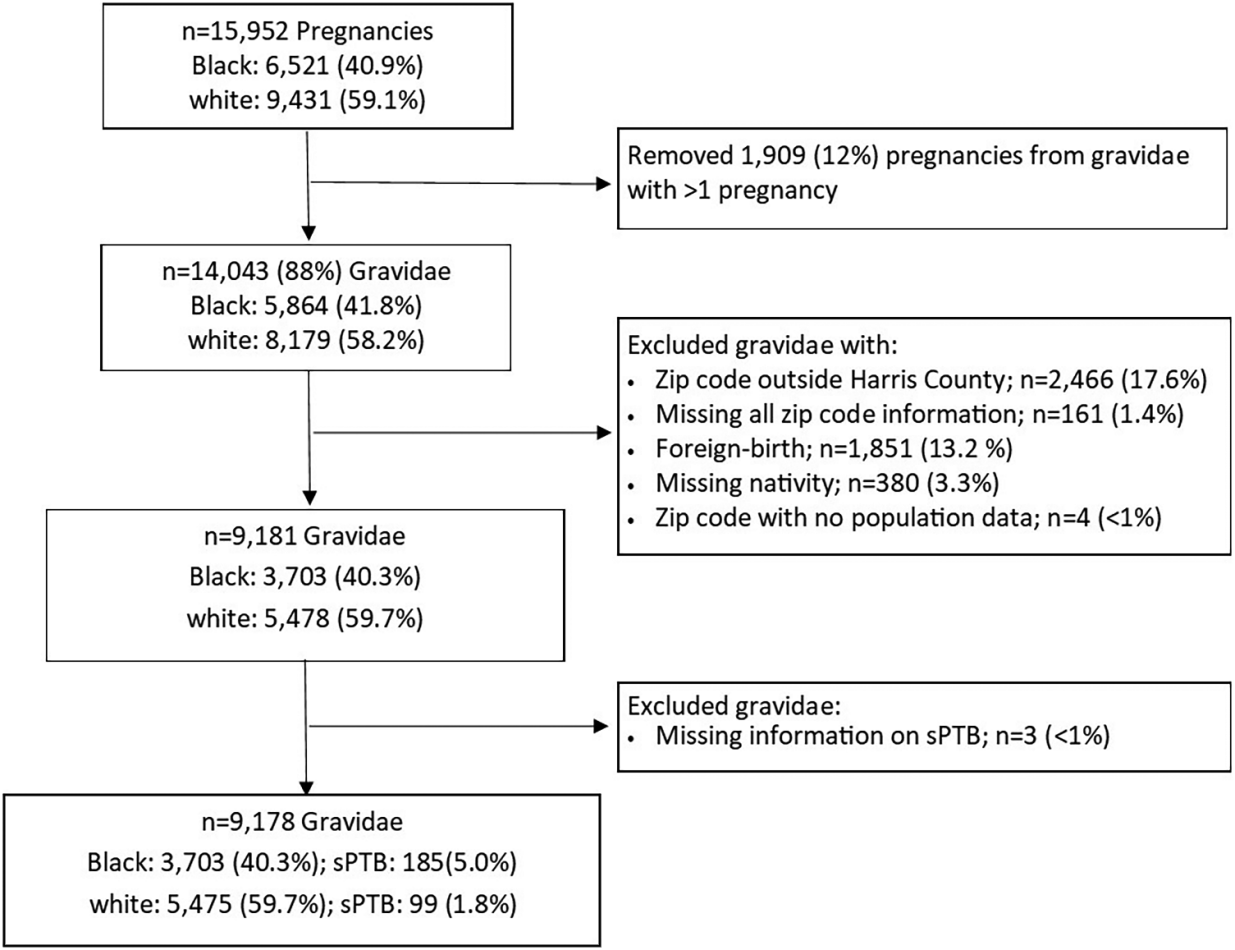
Flow chart of study inclusion among non-Hispanic gravidae in PeriBank (2011–2020).

The present analysis was based on data from self-identified non-Hispanic Black and non-Hispanic white (hereafter referred to as Black and white) gravidae who delivered a singleton live birth with no identified congenital anomaly between August 2011 and December 2020. If gravidae had more than one eligible pregnancy, we randomly selected one pregnancy for the present analysis resulting in 14,043 pregnancies from 5,864 (41.8%) Black and 8,179 (58.2%) white gravidae. We then assessed eligibility based on residence in Harris County using the self-reported zip code of domicile residence in the 2nd/3rd trimester. If this zip code was missing, we relied on the 1st trimester (*n* = 22) or the preconception (*n* = 4) zip code. We excluded 2,466 (17.6%) gravidae with reported zip codes outside of Harris County and 161 (1.4%) gravidae who were missing information on all residential zip codes. We further excluded 1,851 (13.2%) gravidae born outside the U.S. and those missing nativity information (*n* = 380; 3.3%), resulting in 9,181 gravidae (Figure 1).

### 2.2. Neighborhood-level EJ burden and socioeconomic deprivation

To evaluate the impact of living in EJ communities, we utilized U.S. Environmental Protection Agency's (EPA's) Environmental Justice Screening and Mapping Tool (EJScreen) to generate zip-code level EJ indices for ozone, fine particulate matter (PM_2.5_), and proximity to National Priority List (NPL) sites (i.e., Superfund sites) for Harris County (27). An EJ index combines area-level information for both the specific environmental exposure (e.g., ozone) and population characteristics (e.g., percentages of low income and persons of color). Hence, each EJ index provides a measure of pollution burden due to a particular environmental contaminant or source through a social equity lens. EJ indices are highest in areas where there is both a large pollution burden and high proportion of socioeconomically disadvantaged individuals and are represented as percentile rankings (ranging from 0 to 100) relative to data from the entire state of Texas. The zip code-level EJ indices for ozone, PM_2.5_, and proximity to NPL sites were linked to gravidae based on their reported zip code of residence. We excluded four gravidae from two zip codes with no population data (Figure 1).

We also computed the area deprivation index (ADI), a composite metric of 17 indicators from the U.S. Census (28). Higher values of ADI indicate more socioeconomically deprived areas. We obtained zip code tabulation area (ZCTA)-level data from the American Community Survey 5-year estimates (2014–2018) to calculate ADI for all ZCTAs in Harris County and assigned ADI scores to each participant in our study using a ZCTA-to-zip code crosswalk.

### 2.3. Spontaneous preterm birth

We obtained an indicator of whether a participant experienced spontaneous preterm birth (sPTB; i.e., delivery of an infant prior to 37 weeks of gestation and resulting from preterm premature rupture of the membranes or spontaneous labor) from PeriBank records. Gravidae with indicated preterm birth were excluded from this analysis. Three gravidae (<1%) were missing this outcome information (Figure 1).

### 2.4. Covariates

We abstracted several covariates from PeriBank records for each study participant including: maternal age (continuous), parity (0, 1, ≥ 2), maternal education (less than a college degree, college degree or higher), marital status (married, unmarried), alcohol consumption (ever, never), insurance (private, other), pre-pregnancy BMI (<25, 25–30, ≥ 30 kg/m^2^), smoking (ever, never), adequacy of prenatal care (inadequate/intermediate, adequate, adequate plus) (29). We also calculated a binary variable indicating whether a participant had a history of any of the following previous pregnancy complications: preterm birth, intrauterine growth restriction, macrosomia, stillbirth, preeclampsia, congenital anomaly, gestational diabetes, preterm premature rupture of the membranes, hemorrhage, endometriosis, placental abruption or placenta previa, chorioamnionitis, or oligohydramnios.

### 2.5. Statistical analysis

We conducted descriptive statistics for the study population as a whole and separately among Black and white gravidae. To explore the impact of ADI and EJ indices on Black-White disparities in the occurrence of sPTB, we conducted a series of complete case logistic regression models using generalized estimating equations to account for clustering among gravidae residing in the same zip code. First, we assessed the crude association between race (Black vs. white) and sPTB (Model 1) to quantify the extent of the Black-White disparity. We then added maternal age, insurance, alcohol use, marital status, adequacy of prenatal care, and history of pregnancy complications) to Model 2 to evaluate the combined contributions of these individual-level sociodemographic and medical characteristics to the Black-White disparity in preterm birth. These covariates were included based on *a priori* knowledge regarding their association with sPTB. Finally, to evaluate whether neighborhood-level factors further attenuated this disparity, we separately included each EJ Index or ADI in Model 2. A comparison of the odds ratio (OR) describing the Black-White disparity in sPTB between models with or without each neighborhood-level variable informs the extent to which each factor contributes to the observed racial disparity. In addition to this approach, which utilizes a mediation framework to evaluate the contribution of neighborhood-level factors on racial disparities, we conducted stratified analyses (adjusted for the same set of covariates as Model 2) to explore whether race modifies associations of neighborhood-level factors with sPTB (30). In all models, the EJ index or ADI was modeled continuously. In the race-stratified models, ORs and 95% confidence intervals are presented based on a 10-unit increase in each neighborhood-level metric.

As a sensitivity analysis, we repeated all analyses excluding variables for adequacy of prenatal care and history of pregnancy complications because they have the potential to mediate associations between neighborhood-level factors and adverse pregnancy outcomes. We then made comparisons to the full models to evaluate whether they obscured the impact of EJ burden or neighborhood-level socioeconomic deprivation on Black-White disparities in sPTB. All analyses were conducted using SAS (version 9.4, SAS Institute Inc., Cary, NC, USA).

## 3. Results

Our study included 9,178 gravidae: 3,703 (40.3%) Black gravidae and 5,475 (59.7%) white gravidae (Figure 1). Table 1 highlights several differences between these two groups with Black gravidae generally experiencing greater disadvantage than white gravidae. The prevalence of sPTB was 3.1% overall and was more than twice among Black gravidae than among white gravidae (5.0% vs. 1.8%). Compared with their white counterparts, fewer Black gravidae were married (46.3% vs. 91.7%), held at least a college degree (28.5% vs. 78.6%) or had private insurance (24.6% vs. 82.5%). A greater proportion of Black compared with white gravidae had a pre-pregnancy BMI ≥30 kg/m^2^ (32.1% vs. 14.8%) or were classified as having received inadequate/intermediate prenatal care (31.8% vs. 9.5%). With the exception of pre-pregnancy BMI and adequacy of prenatal care, the proportion of missing observations for each variable was <5%. The final analytic sample size in our study was 8,086 gravidae, including 121 sPTB among Black gravidae and 80 sPTB among white gravidae.

**Table 1:** Individual-level maternal sociodemographic characteristics of 9,178 US-born non-Hispanic white and Black gravidae with singleton livebirths in Harris County, Texas, PeriBank (2011–2020).

	All Gravidae (*n* = 9,178) *n* (%)	White Gravidae (*n* = 5,475) *n* (%)	Black Gravidae (*n* = 3,703) *n* (%)
Age			
Mean ± SD	29.8 ± 5.6	31.4 ± 4.7	28.5 ± 6.2
Missing	11 (0.1)	7 (0.1)	4 (0.1)
Pre-pregnancy BMI (kg/m^2^)			
<25	4,505 (49.1)	3,284 (60.0)	1,221 (33.0)
25–30	2,002 (21.8)	1,146 (20.9)	856 (23.1)
≥30	1,995 (21.7)	808 (14.8)	1,187 (32.1)
Missing	676 (7.4)	237 (4.3)	439 (11.9)
Parity			
0	4,422 (48.1)	2,716 (49.6)	1,706 (46.1)
1	2,861 (31.2)	1,813 (33.1)	1,048 (28.3)
≥2	1,886 (20.6)	941 (17.2)	945 (25.5)
Missing	9 (0.1)	5 (0.1)	4 (0.1)
Married			
No	2,389 (26.0)	432 (7.9)	1,957 (52.9)
Yes	6,731 (73.3)	5,018 (91.7)	1,713 (46.3)
Missing	58 (0.6)	25 (0.5)	33 (0.9)
Highest level of education			
<College degree	3,521 (38.4)	1,020 (18.6)	2,501 (67.5)
College degree or higher	5,355 (58.4)	4,301 (78.6)	1,054 (28.5)
Missing	302 (3.3)	154 (2.8)	148 (4.0)
Smoking			
Ever	2,032 (22.1)	1,263 (23.1)	769 (20.8)
Never	7,141 (77.8)	4,209 (76.9)	2,932 (79.2)
Missing	5 (0.1)	3 (0.1)	2 (0.1)
Alcohol Consumption			
Ever	6,854 (74.7)	4,518 (82.5)	2,336 (63.1)
Never	2,318 (25.3)	954 (17.4)	1,364 (36.8)
Missing	6 (0.1)	3 (0.1)	3 (0.1)
Insurance			
Private	5,429 (59.2)	4,519 (82.5)	910 (24.6)
Other	3,484 (38.0)	814 (14.9)	2,670 (72.1)
Missing	265 (2.9)	142 (2.6)	123 (3.3)
Adequacy of prenatal care			
Inadequate/intermediate	1,695 (18.5)	518 (9.5)	1,177 (31.8)
Adequate	3,727 (40.6)	2,651 (48.4)	1,076 (29.1)
Adequate plus	2, 927 (31.9)	1,950 (35.6)	977 (26.4)
Missing	829 (9.0)	356 (6.5)	473 (12.8)
Previous pregnancy complications			
Yes	3,110 (33.9)	1,747 (31.9)	1,363 (36.8)
No	6,066 (66.1)	3,728 (68.1)	2,338 (63.1)
Missing	2 (0.02)	0 (0.0)	2 (0.04)
Spontaneous Preterm Birth			
Yes	284 (3.1)	99 (1.8)	185 (5.0)
No	8,894 (96.9)	5,376 (98.2)	3,518 (95.0)

**Figure 2:**
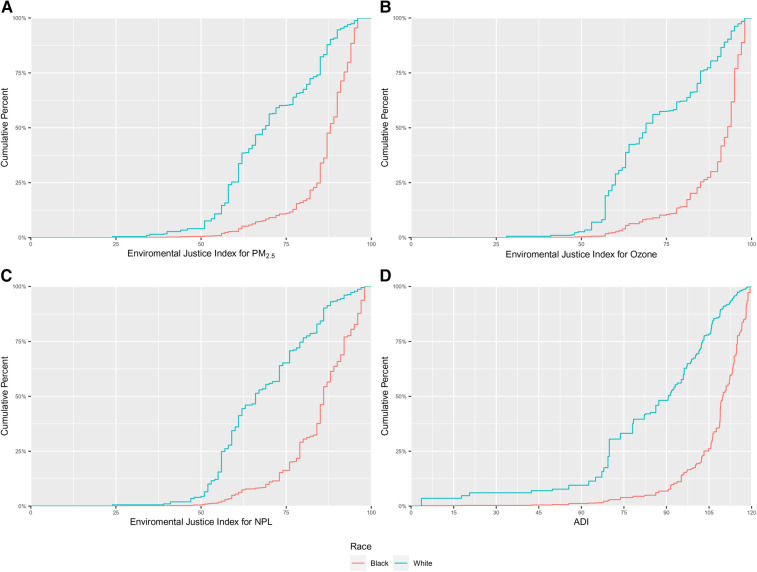
Cumulative distributions of (**A**) environmental justice (EJ) index for fine particulate matter (PM_2.5_); (**B**) EJ index for ozone; (**C**) EJ index for national priority list (NPL) sites; and, (**D**) area deprivation index (ADI) among 8,086 US-born non-Hispanic Black and white gravidae in Harris County, Texas, PeriBank (2011–2020).

Among women included in our analytic sample, we observed that the neighborhoods in which Black gravidae live were characterized by greater EJ burden and sociodemographic deprivation, as demonstrated in Figure 2, displaying the cumulative distribution functions for each of the neighborhood-level variables, by race. In all cases, there was a shift in the distribution of values for the neighborhood-level indicator towards higher values among Black as compared with white gravidae. The distributions of neighborhood-level factors are also presented in Supplementary Table S1. The median and interquartile range (IQR; 25%, 75%) for the EJ Index for PM_2.5_ was 88 (85, 92) for Black and 69 (59, 85) for white gravidae, respectively. The race-specific distributions of the EJ Index for ozone were similar to that of the EJ Index for PM_2.5_. The median EJ Index for NPL sites was 86 (79, 92) among Black and 66 (57, 80) among white gravidae. The median (IQR) of ADI was 109.8 (103.2, 115) and 90.7 (69.8, 102.8) for Black and white gravidae, respectively. The spread in the distribution of values for each neighborhood-level variable was much smaller for Black than for white gravidae as evidenced by the relatively narrow interquartile ranges and steep rise of the cumulative distribution functions, particularly for the EJ PM_2.5_ and ozone indices.

### 3.1. Evaluation of EJ burden and ADI as mediators of Black-White disparities in sPTB

Overall, we found more than twice the odds of sPTB among Black compared with white gravidae [Table 2, Model 1: OR = 2.46, 95% CI = 1.89, 3.19]. Adjusting for individual-level sociodemographic variables attenuated, but did not diminish, this disparity (Table 2, Model 2: OR = 1.79, 95% CI = 1.32, 2.44). The Black-White disparity in sPTB after accounting for individual-level variables was only moderately attenuated when further accounting for EJ burden of PM_2.5_ (OR = 1.68, 95% CI = 1.21, 2.32), ozone (OR = 1.68, 95% CI = 1.22, 2.32), NPL sites (OR = 1.69, 95% CI = 1.25, 2.29) or ADI (OR = 1.58, 95% CI = 1.14, 2.18).

**Table 2:** Odds ratios describing the Black-White disparity in spontaneous preterm birth among 8,086 US-born non-Hispanic Black and white gravidae in Harris County, Texas, PeriBank (2011–2020).

	OR (95% CI)
Model 1^a^	2.46 (1.89, 3.19)
Model 2^b^	1.79 (1.32, 2.44)
Model 2^b^ + EJ index for PM_2.5_	1.68 (1.21, 2.32)
Model 2^b^ + EJ index for ozone	1.68 (1.22, 2.32)
Model 2^b^ + EJ index for NPL sites	1.69 (1.25, 2.29)
Model 2^b^ + ADI	1.58 (1.14, 2.18)

ADI, area deprivation index; CI, confidence interval; EJ, environmental justice; NPL, national priorities list; OR, odds ratio; PM_2.5_, fine particulate matter.

^a^ Crude association between race (Black vs. white) and spontaneous preterm birth.

^b^ Association between race (Black and white) and spontaneous preterm birth adjusted age, insurance, alcohol use, marital status, adequacy of prenatal care, and previous pregnancy complications.

### 3.2. Race as a modifier of associations between EJ burden or ADI and sPTB

In the race-stratified analyses (Table 3**)** we observed an increased odds of sPTB in association with each EJ index among Black gravidae and no association between each EJ index and sPTB among white gravidae. The ORs describing the association of a 10-unit increase in the neighborhood-level EJ Index for PM_2.5_ with sPTB was 1.17 (95% CI = 0.97, 1.40) for Black and 1.02 (95% CI = 0.86, 1.21) for white gravidae. Similar associations were observed between the EJ indices for ozone and NPL sites with sPTB. The association between living in a neighborhood with a higher ADI (i.e., a more socioeconomically deprived neighborhood) was the same for both racial groups (Black: OR = 1.11, 95% CI = 0.95, 1.30; white: OR = 1.11, 95% CI = 0.98, 1.25).

**Table 3:** Associations^a^ between EJ indices and ADI and spontaneous preterm birth among 8,086 US-born non-Hispanic Black and white gravidae in harris county, Texas, periBank (2011–2020).

	White Gravidae	Black Gravidae
	OR (95% CI)	OR (95% CI)
EJ index for PM_2.5_	1.02 (0.86, 1.21)	1.17 (0.97, 1.40)
EJ index for ozone	1.01 (0.86, 1.18)	1.18 (0.99, 1.41)
EJ index for NPL sites	0.97 (0.82, 1.15)	1.16 (0.98, 1.39)
ADI	1.11 (0.98, 1.25)	1.11 (0.95, 1.30)

ADI, area deprivation index; CI, confidence interval; EJ, environmental justice; NPL, national priorities list; OR, odds ratio; PM_2.5_, fine particulate matter.

^a^ Adjusted for age, insurance, alcohol use, marital status, adequacy of prenatal care, and previous pregnancy complications.

In our sensitivity analyses, models that did not include prenatal care and previous pregnancy complication produced similar conclusions (Supplementary Tables S2 and S3).

## 4. Discussion

Our study utilized data from an existing and well curated perinatal data repository to explore the role of EJ burden in Black-White disparities in sPTB birth in Harris County, Texas, home to the fourth largest and most diverse city in the U.S.—Houston. We additionally evaluated the role of neighborhood socioeconomic deprivation alone, via ADI. In our study, set in an area characterized by a network of dense, heavily trafficked roadways, many hazardous waste sites, no zoning laws, and the largest seaport in the nation, U.S.-born Black gravidae had substantially greater odds of sPTB compared with U.S.-born white gravidae. Though we found only a modest contribution of neighborhood factors (either EJ burden or ADI) to Black-White disparities in sPTB among women in our study, our analyses suggest racial differences in the magnitude of associations between neighborhood measures of EJ burden and sPTB.

Because individual-level characteristics do not fully explain observed Black-White disparities in preterm birth (8, 9, 11), we must look to other factors, including the neighborhood context, to evaluate their role as key drivers of disparities. While the physiology and timing of initiation of labor persists as largely poorly understood, aspects of the neighborhood environment may increase psychosocial stress experienced by pregnant persons (31). For example, it has been suggested that chronic stress exposures are associated with the release of catecholamines and activation of the HPA-axis, triggering downstream events such as the release of cortisol, which is transported across the placenta (16, 17, 32). While previous reviews provide evidence of the adverse perinatal impact of living in socioeconomically disadvantaged neighborhoods (18, 19), other investigations have attempted to assess the specific contribution of the neighborhood-level socioeconomic context to racial and ethnic health disparities—that is, whether consideration of such factors attenuates the risk of adverse perinatal health outcomes among Black compared with white gravidae. For example, Benmarhnia et al. (10) found zip code-level neighborhood socioeconomic characteristics (i.e., unemployment, poverty, linguistic minority, educational attainment) explained 16.1% of the observed Black-White disparity in preterm birth, nearly equal to the proportion (17.5%) of the disparity explained by individual-level factors (i.e., maternal education, age at delivery, Medicaid enrollee, and missing paternal information).

However, we are aware of only a handful of studies evaluating perinatal health impacts of living in an EJ neighborhood (that is, a neighborhood jointly characterized by increased pollution burden and socioeconomic deprivation) (24, 25, 33–36). In a county-level analysis, Rappazzo et al. (24) reported lower prevalence of preterm birth among counties with poorer overall environmental quality, assessed via a county-level composite index of variables from four environmental domains: air, water, built, and sociodemographic (37). However, the authors also reported differences in the direction and magnitude of associations when they evaluated domain-specific associations and within urban-rural strata (24). Additionally, in a follow-up study, there was evidence of interactions among domain-specific effects on county-level prevalence of preterm birth (36). On the other hand, Martenies et al. (25) constructed a census-tract level exposure index incorporating information relating to air pollution, built environment, and social exposures, and found increased risk of preterm birth among women living in areas with greater combined environmental and social exposures. We are unaware of studies that have explicitly evaluated whether Black-White disparities in perinatal health outcomes are mediated through residence in an EJ neighborhood. Although accounting for EJ burden (or ADI) attenuated the observed Black-White disparity in sPTB in the present study, the attenuation was modest, pointing to potential (as yet) unmeasured determinants of this disparity in our population.

To fully explore how the neighborhood context might influence health disparities, stratified analyses may provide insight into the potential differential impact that living in a disadvantaged context may have among gravidae of different racial groups (30). The only previous study of which we are aware that has evaluated racial differences in the impact of living in an EJ neighborhood is the study by Martenies et al. (25) which included pregnant individuals across the U.S. who were enrolled in the national ECHO Cohort. When stratified by race, the authors found no evidence of an association between a one standard deviation increase in the census tract-level combined cumulative exposure index and risk of preterm birth among white participants (RR = 0.99; 95% CI = 0.95, 1,03) although they report an 8% increased risk of preterm birth among Black participants (RR = 1.08, 95% CI = 1.00, 1.16). In the current study, we also found suggestive evidence that the impact of living in an EJ neighborhood is limited to Black gravidae. Metrics of EJ burden may capture evidence of systemic or structural racial inequities which amplify adverse effects of environmental toxicant exposure amongst Black and other vulnerable and marginalized populations (31).

Interestingly, our evaluation of living in neighborhoods characterized by socioeconomic deprivation (without regards to increased environmental exposure burden) revealed a different pattern of association. In the current study, we observed identical effect estimates representing the association between ADI and sPTB among Black and white gravidae, in contrast to the results of the analyses of EJ Indices, where associations were only observed among Black gravidae. Although the literature is generally supportive of associations between neighborhood socioeconomic deprivation and adverse perinatal health outcomes overall (18, 19), results of previous studies investigating race-specific associations between neighborhood socioeconomic deprivation and preterm birth have been mixed (18–20, 38–40), with some studies indicating larger effect estimates associated with living in a socioeconomically disadvantaged neighborhood among white compared with Black gravidae (19) while others report the opposite (18). Given mixed results, future analyses using data from studies that allow the characterization of specific aspects of the neighborhood context as well as individual-level stressors and buffers may help further inform mechanisms through which the neighborhood environment may (or may not) differentially impact Black and white gravidae and result in disproportionate perinatal health outcomes.

Our study included a relatively large number of births, spanning nine years and including more than 8,000 women. Even so, given the low prevalence of sPTB, our analysis suffered from small numbers. Because there were few cases of sPTB in several strata when data were stratified by both race and categories of exposure, we present associations based on 10-unit increases in each neighborhood factor, which was around a single standard deviation in the distributions of the EJ indices and less than 1 standard deviation in the distribution of ADI; this approach to analyzing environmental justice burden variables is also similar to that utilized in a previous investigation (25). However, it is possible that the relation between living in a deprived neighborhood and sPTB would be better characterized through a comparison of gravidae who live in areas with relatively higher vs. lower socioeconomic deprivation or environmental justice burden. Interestingly, the distributions of indices representing environmental justice burden due to PM_2.5_, ozone, and NPL sites were very similar and thus, it is likely that these metrics were all measuring generalized EJ burden. It is also possible that the neighborhood measures we used did not fully capture the scope of EJ burden or socioeconomic deprivation experienced by gravidae in our study, in part due to the exposure misclassification resulting from the relatively large geographic area covered by zip codes. In contrast, smaller geographical units would better capture dimensions of the neighborhood context. Although our analysis was constrained to the use of zip codes to define participant's neighborhood environment, we recommend future studies to further investigate the present findings using data sources that allow for a more spatially resolved neighborhood assessment.

In this study of retrospectively collected data from a large number of U.S.-born gravidae living in the third most populous county in the U.S. with myriad sources of environmental exposure and demonstrated environmental injustice, we observed clear evidence of Black-White disparities in sPTB, an outcome with immense public health implications. Though we observed only moderate evidence of the contribution of living in EJ neighborhoods to this disparity, our study suggests living in an EJ neighborhood may differentially impact Black and white gravidae.

## Data Availability

The data analyzed in this study is subject to the following licenses/restrictions: PeriBank data are available for any investigator to access with appropriate approvals by the PeriBank Governing Board and their local Institutional Review Board. Requests to access these datasets should be directed to Jia Chen (jiac@bcm.edu).

## References

[1] Waitzman NJ Jalali A Grosse SD. Preterm birth lifetime costs in the United States in 2016: an update. Semin Perinatol. (2021) 45(3):151390. 10.1016/j.semperi.2021.151390 33541716 PMC10549985

[2] Wu P Gulati M Kwok CS Wong CW Narain A O’Brien S Preterm delivery and future risk of maternal cardiovascular disease: a systematic review and meta-analysis. J Am Heart Assoc. (2018) 7(2):e007809. 10.1161/JAHA.117.007809 29335319 PMC5850169

[3] McNestry C Killeen SL Crowley RK McAuliffe FM. Pregnancy complications and later life women’s health. Acta Obstet Gynecol Scand. (2023) 102(5):523–31. 10.1111/aogs.14523 36799269 PMC10072255

[4] Luu TM Katz SL Leeson P Thébaud B Nuyt AM. Preterm birth: risk factor for early-onset chronic diseases. CMAJ. (2016) 188(10):736–46. 10.1503/cmaj.150450 26644500 PMC4938684

[5] Raju TNK Buist AS Blaisdell CJ Moxey-Mims M Saigal S. Adults born preterm: a review of general health and system-specific outcomes. Acta Paediatr. (2017) 106(9):1409–37. 10.1111/apa.13880 28419544

[6] Martin JA Hamilton BE Osterman MJK Driscoll AK. Births: final data for 2018. Natl Vital Stat Rep. (2019) 68(13):1–47. 32501202

[7] Montoya-Williams D Barreto A Fuentes-Afflick E Collins JW Jr. Nativity and perinatal outcome disparities in the United States: beyond the immigrant paradox. Semin Perinatol. (2022) 46(8):151658. 10.1016/j.semperi.2022.151658 36137831 PMC10016119

[8] Thoma ME Drew LB Hirai AH Kim TY Fenelon A Shenassa ED. Black-White disparities in preterm birth: geographic, social, and health determinants. Am J Prev Med. 2019;57(5):675–86. 10.1016/j.amepre.2019.07.007 31561920

[9] DeSisto CL Hirai AH Collins JW Jr Rankin KM. Deconstructing a disparity: explaining excess preterm birth among U.S.-born black women. Ann Epidemiol. (2018) 28(4):225–30. 10.1016/j.annepidem.2018.01.012 29433978

[10] Benmarhnia T Huang J Basu R Wu J Bruckner TA. Decomposition analysis of Black-White disparities in birth outcomes: the relative contribution of air pollution and social factors in California. Environ Health Perspect. (2017) 125(10):107003. 10.1289/EHP490 28977781 PMC5933346

[11] Su D Samson K Hanson C Anderson Berry AL Li Y Shi L Racial and ethnic disparities in birth outcomes: a decomposition analysis of contributing factors. Prev Med Rep. (2021) 23:101456. 10.1016/j.pmedr.2021.101456 34285869 PMC8273196

[12] Lhila A Long S. What is driving the black-white difference in low birthweight in the US? Health Econ. (2012) 21(3):301–15. 10.1002/hec.1715 21294220

[13] Braveman P Dominguez TP Burke W Dolan SM Stevenson DK Jackson FM Explaining the Black-White disparity in preterm birth: a consensus statement from a multi-disciplinary scientific work group convened by the march of dimes. Front Reprod Health. (2021) 3:684207. 10.3389/frph.2021.684207 36303973 PMC9580804

[14] Krieger N. Methods for the scientific study of discrimination and health: an ecosocial approach. Am J Public Health. (2012) 102(5):936–44. 10.2105/AJPH.2011.300544 22420803 PMC3484783

[15] McEwen BS Tucker P. Critical biological pathways for chronic psychosocial stress and research opportunities to advance the consideration of stress in chemical risk assessment. Am J Public Health. (2011) 101(Suppl 1):S131–9. 10.2105/AJPH.2011.300270 22021312 PMC3222511

[16] Hobel C Culhane J. Role of psychosocial and nutritional stress on poor pregnancy outcome. J Nutr. (2003) 133(5 Suppl 2):1709S–17S. 10.1093/jn/133.5.1709S 12730488

[17] Mulder EJ Robles de Medina PG Huizink AC Van den Bergh BR Buitelaar JK Visser GH. Prenatal maternal stress: effects on pregnancy and the (unborn) child. Early Hum Dev. (2002) 70(1–2):3–14. 10.1016/S0378-3782(02)00075-0 12441200

[18] Mutambudzi M Meyer JD Reisine S Warren N. A review of recent literature on materialist and psychosocial models for racial and ethnic disparities in birth outcomes in the US, 2000–2014. Ethn Health. (2017) 22(3):311–32. 10.1080/13557858.2016.1247150 27852109

[19] Ncube CN Enquobahrie DA Albert SM Herrick AL Burke JG. Association of neighborhood context with offspring risk of preterm birth and low birthweight: a systematic review and meta-analysis of population-based studies. Soc Sci Med. (2016) 153:156–64. 10.1016/j.socscimed.2016.02.014 26900890 PMC7302006

[20] Phillips GS Wise LA Rich-Edwards JW Stampfer MJ Rosenberg L. Neighborhood socioeconomic status in relation to preterm birth in a U.S. Cohort of black women. J Urban Health. (2013) 90(2):197–211. 10.1007/s11524-012-9739-x 22752302 PMC3675720

[21] O’Campo P Burke JG Culhane J Elo IT Eyster J Holzman C Neighborhood deprivation and preterm birth among non-hispanic black and white women in eight geographic areas in the United States. Am J Epidemiol. (2008) 167(2):155–63. 10.1093/aje/kwm277 17989062

[22] Song S Gao Z Zhang X Zhao X Chang H Zhang J Ambient fine particulate matter and pregnancy outcomes: an umbrella review. Environ Res. (2023) 235:116652. 10.1016/j.envres.2023.116652 37451569

[23] Hung TH Chen PH Tung TH Hsu J Hsu TY Wan GH. Risks of preterm birth and low birth weight and maternal exposure to NO(2)/PM(2.5) acquired by dichotomous evaluation: a systematic review and meta-analysis. Environ Sci Pollut Res Int. (2023) 30(4):9331–49. 10.1007/s11356-022-24520-5 36474040

[24] Rappazzo KM Messer LC Jagai JS Gray CL Grabich SC Lobdell DT. The associations between environmental quality and preterm birth in the United States, 2000-2005: a cross-sectional analysis. Environ Health. (2015) 14:50. 10.1186/s12940-015-0038-3 26051702 PMC4464856

[25] Martenies SE Zhang M Corrigan AE Kvit A Shields T Wheaton W Associations between combined exposure to environmental hazards and social stressors at the neighborhood level and individual perinatal outcomes in the ECHO-wide cohort. Health Place. (2022) 76:102858. 10.1016/j.healthplace.2022.102858 35872389 PMC9661655

[26] Antony KM Hemarajata P Chen J Morris J Cook C Masalas D Generation and validation of a universal perinatal database and biospecimen repository: periBank. J Perinatol. (2016) 36(11):921–9. 10.1038/jp.2016.130 27629376

[27] U.S. Environmental Protection Agency. EJScreen: Environmental Justice Screen and Mapping Tool. Available at: https://www.epa.gov/ejscreen (Updated June 26, 2023).

[28] Singh GK. Area deprivation and widening inequalities in US mortality, 1969-1998. Am J Public Health. (2003) 93(7):1137–43. 10.2105/AJPH.93.7.1137 12835199 PMC1447923

[29] Kotelchuck M. The adequacy of prenatal care utilization index: its US distribution and association with low birthweight. Am J Public Health. (1994) 84(9):1486–9. 10.2105/AJPH.84.9.1486 8092377 PMC1615176

[30] Burris HH Valeri L James-Todd T. Statistical methods to examine contributors to racial disparities in perinatal outcomes. Semin Perinatol. (2022) 46(8):151663. 10.1016/j.semperi.2022.151663 36180264

[31] Gee GC Payne-Sturges DC. Environmental health disparities: a framework integrating psychosocial and environmental concepts. Environ Health Perspect. (2004) 112(17):1645–53. 10.1289/ehp.7074 15579407 PMC1253653

[32] Hobel CJ Goldstein A Barrett ES. Psychosocial stress and pregnancy outcome. Clin Obstet Gynecol. (2008) 51(2):333–48. 10.1097/GRF.0b013e31816f2709 18463464

[33] Alcala E Brown P Capitman JA Gonzalez M Cisneros R. Cumulative impact of environmental pollution and population vulnerability on pediatric asthma hospitalizations: a multilevel analysis of CalEnviroScreen. Int J Environ Res Public Health. (2019) 16(15):2683. 10.3390/ijerph16152683 31357578 PMC6696276

[34] Krajewski AK Rappazzo KM Langlois PH Messer LC Lobdell DT. Associations between cumulative environmental quality and ten selected birth defects in Texas. Birth Defects Res. (2021) 113(2):161–72. 10.1002/bdr2.1788 32864854 PMC8091812

[35] Patel AP Jagai JS Messer LC Gray CL Rappazzo KM Deflorio-Barker SA Associations between environmental quality and infant mortality in the United States, 2000–2005. Arch Public Health. (2018) 76:60. 10.1186/s13690-018-0306-0 30356923 PMC6191999

[36] Grabich SC Rappazzo KM Gray CL Jagai JS Jian Y Messer LC Additive interaction between heterogeneous environmental quality domains (air, water, land, sociodemographic, and built environment) on preterm birth. Front Public Health. (2016) 4:232. 10.3389/fpubh.2016.00232 27822465 PMC5076290

[37] Messer LC Jagai JS Rappazzo KM Lobdell DT. Construction of an environmental quality index for public health research. Environ Health. (2014) 13(1):39. 10.1186/1476-069X-13-39 24886426 PMC4046025

[38] Messer LC Kaufman JS Dole N Savitz DA Laraia BA. Neighborhood crime, deprivation, and preterm birth. Ann Epidemiol. (2006) 16(6):455–62. 10.1016/j.annepidem.2005.08.006 16290179

[39] Messer LC Vinikoor LC Laraia BA Kaufman JS Eyster J Holzman C Socioeconomic domains and associations with preterm birth. Soc Sci Med. (2008) 67(8):1247–57. 10.1016/j.socscimed.2008.06.009 18640759

[40] Braveman PA Heck K Egerter S Marchi KS Dominguez TP Cubbin C The role of socioeconomic factors in Black-White disparities in preterm birth. Am J Public Health. (2015) 105(4):694–702. 10.2105/AJPH.2014.302008 25211759 PMC4358162

