# Supplementary material for: Environmental justice burden and Black-White disparities in spontaneous preterm birth in Harris County, Texas
Source: Front Reprod Health. 2023 Dec 19;5:1296590. doi: 10.3389/frph.2023.1296590 (PMC10766384; doi:10.3389/frph.2023.1296590)
Supplement: Supplementary file 1 [file Datasheet1.docx]

**Supplemental Information**

| Supplemental Table 1 Distribution of neighborhood-level variables among 8,086 non-Hispanic Black and white gravidae in Harris County, Texas, PeriBank (2011 –2020) | | | | | | | | | | | | | | |
| --- | --- | --- | --- | --- | --- | --- | --- | --- | --- | --- | --- | --- | --- | --- |
|  | **All Gravidae (n=8,086)** | | | |  | **Black Gravidae (n=3,107)** | | | |  | **White Gravidae (n=4,979)** | | | |
|  | **PM_2.5_** | **Ozone** | **NPL** | **ADI** |  | **PM_2.5_** | **Ozone** | **NPL** | **ADI** |  | **PM_2.5_** | **Ozone** | **NPL** | **ADI** |
| mean (sd) | 76.3 (14.8) | 78.8 (15.5) | 74.6 (15.0) | 93.1 (24.2) |  | 86.0 (9.6) | 89.1 (10.3) | 84.5 (11.0) | 107.2 (13.0) |  | 70.3 (14.2) | 72.4  (14.8) | 68.5 (13.9) | 84.3 (25.4) |
| Min | 24 | 28 | 24 | 3.6 |  | 24 | 28 | 24 | 3.6 |  | 24 | 28 | 24 | 3.6 |
| 10% | 57 | 57 | 55 | 67.2 |  | 72 | 73 | 70 | 92.7 |  | 54 | 57 | 52 | 62.6 |
| 25% | 62 | 63 | 61 | 78.4 |  | 85 | 85 | 79 | 103.2 |  | 59 | 60 | 57 | 69.8 |
| 50% | 82 | 84 | 76 | 100.6 |  | 88 | 93 | 86 | 109.8 |  | 69 | 69 | 66 | 90.7 |
| 75% | 88 | 93 | 86 | 110 |  | 92 | 95 | 92 | 115 |  | 85 | 85 | 80 | 102.8 |
| 90% | 93 | 96 | 94 | 115.6 |  | 95 | 98 | 97 | 118.1 |  | 88 | 93 | 86 | 109.7 |
| max | 96 | 98 | 98 | 119.5 |  | 96 | 98 | 98 | 119.5 |  | 96 | 98 | 98 | 119.5 |

Note: ADI: area deprivation index; EJ: environmental justice; NPL: national priorities list; PM_2.5_: fine particulate matter

| **Supplemental Table 2.** Odds ratios describing the Black-White disparity in spontaneous preterm birth among 8,086 US-born non-Hispanic Black and white gravidae in Harris County, Texas, PeriBank (2011–2020) | | |
| --- | --- | --- |
|  | OR (95% CI) | |
| Model 1^a^ | 2.46 (1.89, 3.19) | |
| Model 2^b^ | 1.97 (1.44, 2.71) | |
| Model 2^b^ + EJ Index for PM_2.5_ | 1.86 (1.33, 2.61) | |
| Model 2^b^ + EJ Index for Ozone | 1.87 (1.34, 2.60) | |
| Model 2^b^ + EJ Index for NPL Sites | 1.88 (1.37, 2.56) | |
| Model 2^b^ + ADI | 1.72 (1.24, 2.39) | |
| Note: ADI: area deprivation index; CI: confidence interval; EJ: environmental justice; NPL: national priorities list; OR: odds ratio; PM_2.5_: fine particulate matter | |  |
| ^b^Crude association between race (Black vs. white) and spontaneous preterm birth | |  |
| ^c^Association between race (Black vs. white) adjusted for age, insurance, alcohol use, and marital status | |  |

| **Supplemental Table 3**. Associations^a^ between EJ indices and ADI and spontaneous preterm birth among 8,086 US-born non-Hispanic Black and white gravidae in Harris County, Texas, PeriBank (2011–2020) | | | |
| --- | --- | --- | --- |
|  | White Gravidae |  | Black Gravidae |
|  | OR (95% CI) |  | OR (95% CI) |
|  |  |  |  |
| EJ Index for PM_2.5_ | 1.00 (0.85, 1.18) |  | 1.17 (0.98, 1.40) |
| EJ Index for ozone | 0.98 (0.84, 1.15) |  | 1.15 (0.95, 1.39) |
| EJ Index for NPL Sites | 0.95 (0.81, 1.13) |  | 1.14 (0.96, 1.36) |
| ADI | 1.11 (0.98, 1.25) |  | 1.08 (0.87, 1.35) |

Note: ADI: area deprivation index; CI: confidence interval; EJ: environmental justice; NPL: national priorities list; OR: odds ratio; PM_2.5_: fine particulate matter

^a^Adjusted for age, insurance, alcohol use, and marital status
